# Quantification of bactericidal activity using the PATHFAST TB LAM Ag assay during the first 14 days of pulmonary tuberculosis treatment

**DOI:** 10.3389/frabi.2025.1574688

**Published:** 2025-05-15

**Authors:** Ayumi Akinaga, Andreas H. Diacon, Remous Ocloo, Atsushi Yanagida, Naofumi Yoda, Masanori Kawasaki, Yongge Liu

**Affiliations:** ^1^ PHC Corporation, Tokyo, Japan; ^2^ TASK, Cape Town, South Africa; ^3^ Otsuka Pharmaceutical Co., Ltd., Tokyo, Japan; ^4^ Otsuka Pharmaceutical Development & Commercialization, Inc., Rockville, MD, United States

**Keywords:** tuberculosis, treatment monitoring, lipoarabinomannan, biomarker, bacterial load, immunoassay

## Abstract

**Background:**

Tuberculosis (TB) remains a global health challenge. Culture-free, rapid, and quantitative biomarkers to monitor treatment response are critical to accelerate development of better TB treatments. The PATHFAST TB LAM Ag assay (PATHFAST-LAM), a desktop chemiluminescent enzyme immunoassay that measures mycobacterial lipoarabinomannan (LAM) in sputum within 1 hour, is a promising candidate for this purpose. This study aimed to assess whether the PATHFAST-LAM can serve as a rapid, reliable biomarker for monitoring early treatment response in pulmonary TB.

**Methods:**

We conducted a retrospective longitudinal repository study using stored sputum samples from 14-day early bactericidal activity trials involving 75 pulmonary TB patients who received one of five different regimens. The results were compared with those from the TB LAM ELISA “Otsuka” (LAM-ELISA), which was previously shown to measure early bactericidal activity but takes more than 5 hours to obtain results, and conventional culture-based methods.

**Results:**

The LAM concentrations in both raw and decontaminated sputum showed strong correlations between the PATHFAST-LAM and the LAM-ELISA, with Spearman’s correlation coefficients of 0.975 (95% CI: 0.971 – 0.979) and 0.987 (95% CI: 0.984 – 0.989), respectively. LAM concentrations in raw and decontaminated sputum by the PATHFAST-LAM were also highly correlated with a Spearman coefficient of 0.957 (95% CI: 0.950 – 0.964). Importantly, the LAM concentrations by the PATHFAST-LAM correlated with bacterial loads determined by culture-based methods in all five treatment arms (Spearman’s coefficients: 0.723 – 0.947). Furthermore, the change in LAM levels over the treatment period mirrored the changes in bacterial load. Additionally, culture-based methods often result in missing data due to contamination: in our study, we observed a missing data rate of 9.6% (62/649) on quantifying CFU counts and 4.2% (27/649) on obtaining a valid MGIT TTD, while we obtained a valid LAM value with the PATHFAST-LAM (0/634 in raw samples and 0/637 in decontaminated samples).

**Conclusion:**

Our findings suggest that the PATHFAST-LAM can quantify bactericidal activity in the first 14 days of treatment with a quick turnaround time. The test’s utility to monitor conversion from positive to negative and to predict relapse-free cure compared to culture-based methods should be determined in longer trials.

## Introduction

Despite being curable and preventable, tuberculosis (TB) remains a major global health challenge, with an estimated 10.8 million new cases and 1.25 million deaths worldwide in 2023 (World Health Organization (WHO), 2024). Effective monitoring of treatment progress is essential throughout TB treatment and in the development of new TB drugs. However, current culture-based methods are unable to provide real-time quantification of bacterial load and operationally the use of culture-based methods poses infection risks due to the need to cultivate live bacteria. Moreover, culture-based methods are susceptible to delays or failures due to contamination. This challenge is particularly pronounced in low- and middle-income countries (LMICs), where access to TB culture facilities and adequately trained personnel is limited.

To address the limitation of culture-based methods, alternative biomarkers that can safely, conveniently, and accurately quantify or reflect the number of viable bacilli in a timely manner have become a focus of research (Koele et al., 2023; World Health Organization (WHO), 2023; Gillespie et al., 2024; MacLean et al., 2024). In this context, the World Health Organization (WHO) and the UNITE4TB consortium have developed Target Product Profiles (TPP) for TB treatment monitoring markers. These profiles outline not only the desired sensitivity and specificity for such biomarkers but also emphasize practical aspects like assay ease of use and rapid turnaround time to optimize LMICs (World Health Organization (WHO), 2023; Gillespie et al., 2024).

Lipoarabinomannan (LAM), a major glycolipid in the mycobacterial cell wall, is one such potential biomarker (Heyckendorf et al., 2022). Studies have shown that LAM concentration in sputum measured by the TB LAM ELISA “Otsuka” (LAM-ELISA, Otsuka Pharmaceutical, Tokyo, Japan), correlates with colony forming unit (CFU) counts and time to detection (TTD) in BACTEC MGIT 960 Mycobacterial Detection System (MGIT, Becton, Dickinson and Company, NJ, USA) during treatment. These findings suggest that LAM concentration reflects bacterial loads, and that LAM performs as a pharmacodynamic biomarker (Kawasaki et al., 2019; Jones et al., 2022; Dawson et al., 2023). However, the LAM-ELISA is 96-well based, requires over five hours to process samples, and involves extensive manual handling including multiple steps of plate wash and incubation, making it time-consuming and labor-intensive. Additionally, ELISA necessitates batch processing, requiring multiple samples to be collected and processed simultaneously, which delays results for individual samples.

The PATHFAST TB LAM Ag assay (PATHFAST-LAM, PHC Corporation [previously LSI Medience Corporation], Tokyo, Japan) was developed to address these limitations and is currently available as a CE-IVDR certified product. The PATHFAST-LAM combines a desktop device based on chemiluminescent enzyme immunoassay that can measure LAM with minimal human intervention and within one hour. Unlike ELISA, the PATHFAST system can process one sample at a time, enabling faster turnaround for each test. Additionally, samples are pre-treated with a heating step that extracts LAM but also kills live bacteria, thus eliminating the need for infection control during measurement. The assay has a reported limit of detection (LoD) of 6.67 pg/mL (Akinaga et al., 2024), which is comparable to the LAM-ELISA’s LoD of 8.5 pg/mL (Kawasaki et al., 2019). Using biobank sputum specimens from 100 pretreatment patients, Akinaga et al. reported that the PATHFAST-LAM demonstrated a sensitivity of 88.8% and a specificity of 100% to detect TB when compared to culture-based methods as the reference standard and a strong negative correlation between LAM concentration and MGIT TTD (Akinaga et al., 2024).

While initial evaluations are promising, a critical gap remains in evidence regarding the performance of PATHFAST-LAM for quantifying bacterial load during treatment. In this study, we evaluated the performance of PATHFAST-LAM during an early bactericidal activity trial by analyzing stored raw or decontaminated sputum samples from five groups of pulmonary TB patients treated with different regimens, comparing its results to those from the LAM-ELISA and conventional culture-based methods.

## Materials and methods

### Study design and ethics

This study is a retrospective longitudinal repository study (Level 3 evidence) to evaluate the PATHFAST-LAM performance in monitoring response during the early phase of pulmonary TB treatment. Previous exploratory analysis using pre-treatment samples from a biobank (Akinaga et al., 2024) suggested the potential utility of this assay. Building on that foundation, this study represents an initial validation step, extending the evaluation to longitudinal samples collected during the first 14 days of treatment.

It utilized residual samples and microbiological test results collected during stage 1 of the phase Ib/IIa multiple ascending dose/early bacterial activity (MAD/EBA) study (ClinicalTrial.gov identifier: NCT03678688), conducted by Otsuka Pharmaceutical Development & Commercialization, Inc. (Rockville, MD, USA), and published previously (Dawson et al., 2023). This clinical trial took place at two sites: University of Cape Town Lung Institute (Cape Town, South Africa) and TASK Clinical Research Centre (Cape Town, South Africa). The study included 75 HIV-negative patients aged 18 to 64 years, all newly diagnosed with rifampicin- and isoniazid-susceptible pulmonary TB and sputum smear-positive (Score: 1+ or higher on the IUTLD scale). These patients were randomly assigned to receive a 14 days of anti-TB drug treatment with either quabodepistat (OPC-167832) (3 mg [n = 14], 10 mg [n = 14], 30 mg [n = 14], or 90 mg [n = 17]) or RHEZ (n = 16). Sputum samples were collected overnight on days -2, -1, 2, 4, 6, 8, 10, 12, and 14, with day 0 marking the start of anti-TB treatment.

This study was conducted in accordance with the principles outlined in the Declaration of Helsinki and Good Clinical Practice guidelines. Ethical approval was obtained from the institutional review boards or independent ethics committees of PHC Corporation (formerly LSI Medience Corporation, Approval ID: Shindan/Narita 21-02) and the relevant ethics committees at each site involved in the MAD/EBA study that provided specimens and test results.

### Microbiological tests

The PATHFAST TB LAM Ag assay was conducted using samples collected during the above-mentioned study and previously used for the TB LAM ELISA “Otsuka” measurements and stored at TASK Laboratory (Parow, South Africa).

In the MAD/EBA study, sputum specimens were treated as follows: they were homogenized by magnetic stirring and divided for each test. First, 400 µL of raw sputum samples were used for assessing LAM concentration by the LAM-ELISA (raw sputum). The rest was digested with sputasol and used for assessing colony forming unit (CFU) counts on 7H11 culture medium in duplicate. The remaining samples were decontaminated with NaOH and used for culture in BACTEC MGIT 960 Mycobacterial Detection System and for determining LAM concentrations by the LAM-ELISA (decontaminated sputum). LAM was extracted from each sample according to Kawasaki’s methods (Kawasaki et al., 2019). In brief, each 400 µL of raw sputum or decontaminated sputum sample was mixed with 200 µL of 1.2 M NaOH solution, incubated at 100°C for 20 min, and neutralized with 90 µL of 5 M NaH_2_PO_4_ solution. After the LAM-ELISA assessment, the residual LAM extracts were stocked at -70°C and used for the PATHFAST-LAM.

Before testing with the PATHFAST-LAM, the LAM extracts were thawed, mixed, centrifuged at 3,000 *g* for 5 min at 25°C, and supernatants collected. Then, the supernatants were applied to the PATHFAST-LAM according to Akinaga’s methods (Akinaga et al., 2024), and the LAM concentration were automatically obtained.

### Statistical analysis

First, method comparison between LAM-ELISA and PATHFAST-LAM was performed using the software “Analyze-it” (Method Validation edition, version 5.10.8, Analyze-it Software, Ltd., Leeds, UK). Then, correlation analyses were conducted within PATHFAST-LAM to compare raw sputum and decontaminated sputum. Additionally, Spearman’s rank correlation and regression analyses were performed with Analyse-it to evaluate the associations between CFU and PATHFAST-LAM (raw sputum) for each treatment regimen, as well as between MGIT TTD and PATHFAST-LAM (raw sputum) without adjusting for potential confounders.

To further examine regimen-specific effects and account for within-patient variability, a linear mixed-effects model was constructed using the *lmer* function from the *lme4* R package (version 4.4.2, R Foundation for Statistical Computing, Vienna, Austria) with the following model specification:


$$\Delta_{Log10}CFU_{ij}=\beta_{0}+\beta_{1}\cdot \Delta Log_{10}LAM_{ij}+\beta_{2}\cdot regimen_{j}+\beta_{3}\cdot day_{i}+\beta_{4}\cdot (\Delta Log_{10}LAM_{ij}\times regimen_{j})+\beta 5\cdot (regimen_{j}\times day_{i})+u_{i}+\varepsilon_{ij}$$

where ΔLog_10_ CFU_ij_ represents the change in CFU count for patient i on day j, ΔLog_10_ LAM_ij_ is the corresponding change in LAM concentration, and regimen_j_ is a categorical variable indicating the treatment regimen. The model includes interaction terms to evaluate regimen-specific differences in the association between ΔLog_10_ CFU and ΔLog_10_ LAM, as well as time-dependent effects. A random intercept (u_i_) was included for each patient to account for within-subject correlation.

Fixed-effect estimates and pairwise comparisons of regimen-specific slopes (ΔLog_10_ CFU/ΔLog_10_ LAM) were obtained using estimated marginal trends with the *emtrends* function in the *emmeans* package. Degrees of freedom were calculated using the Kenward-Roger method, and multiple comparisons were adjusted using Tukey’s method. A similar model was applied to analyze the relationship between LAM and MGIT TTD, excluding the 90 mg regimen due to concerns about drug carryover effects.

## Results

This study compared the results from the PATHFAST-LAM with those from previously published results of the LAM-ELISA and culture-based methods (Dawson et al., 2023). Demographics and the baseline characteristics of this study population were the same as those reported previously with the LAM-ELISA and culture results (Dawson et al., 2023), allowing direct comparison with previous study results.

### Correlation between PATHFAST-LAM and LAM-ELISA

**Figure 1:**
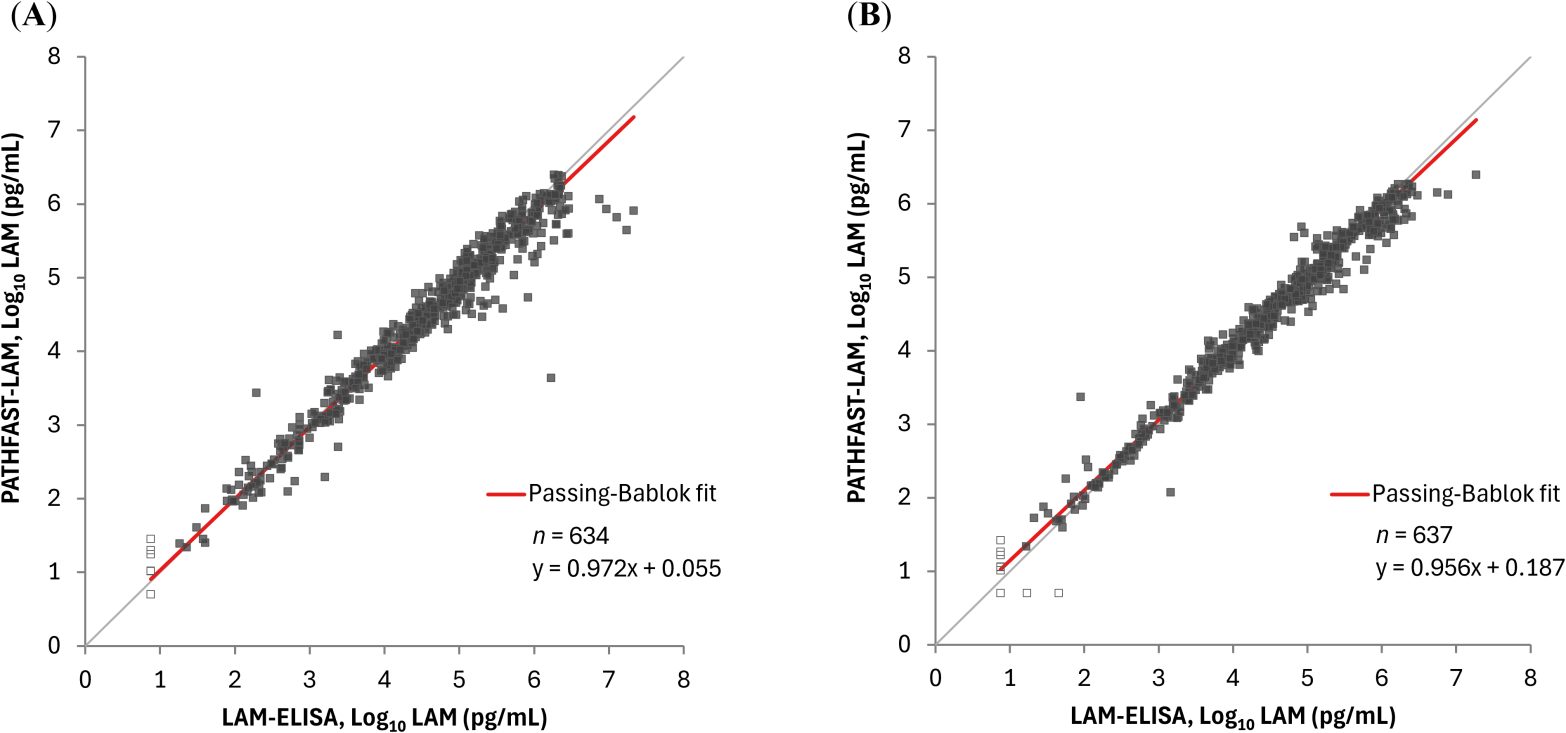
Correlation between sputum LAM concentrations by the PATHFAST-LAM and LAM-ELISA. LAM concentrations below the lower limit of measurement were plotted as half of the lower limit of measurement (Log_10_ 5.00 pg/mL for the PATHFAST-LAM and Log_10_ 7.50 pg/mL for the LAM-ELISA) and represented as open dots. **(A)** Raw sputum. **(B)** Decontaminated sputum.

We assessed the correlation between the PATHFAST-LAM and LAM-ELISA using sputa from all patients and time points. Spearman’s correlation coefficients were 0.975 (95% CI: 0.971 – 0.979) for raw sputum and 0.987 (95% CI: 0.984 – 0.989) for decontaminated sputum. Similarly, the slopes and intercepts were 0.972 (95% CI: 0.959 – 0.985) and 0.055 (95% CI: 0.000 – 0.144) for raw sputum, and 0.956 (95% CI: 0.945 – 0.966) and 0.187 (95% CI: 0.147 – 0.233) for decontaminated sputum (**Figure 1**).

### Correlation between LAM concentrations in raw sputum and decontaminated sputum by the PATHFAST-LAM

**Figure 2:**
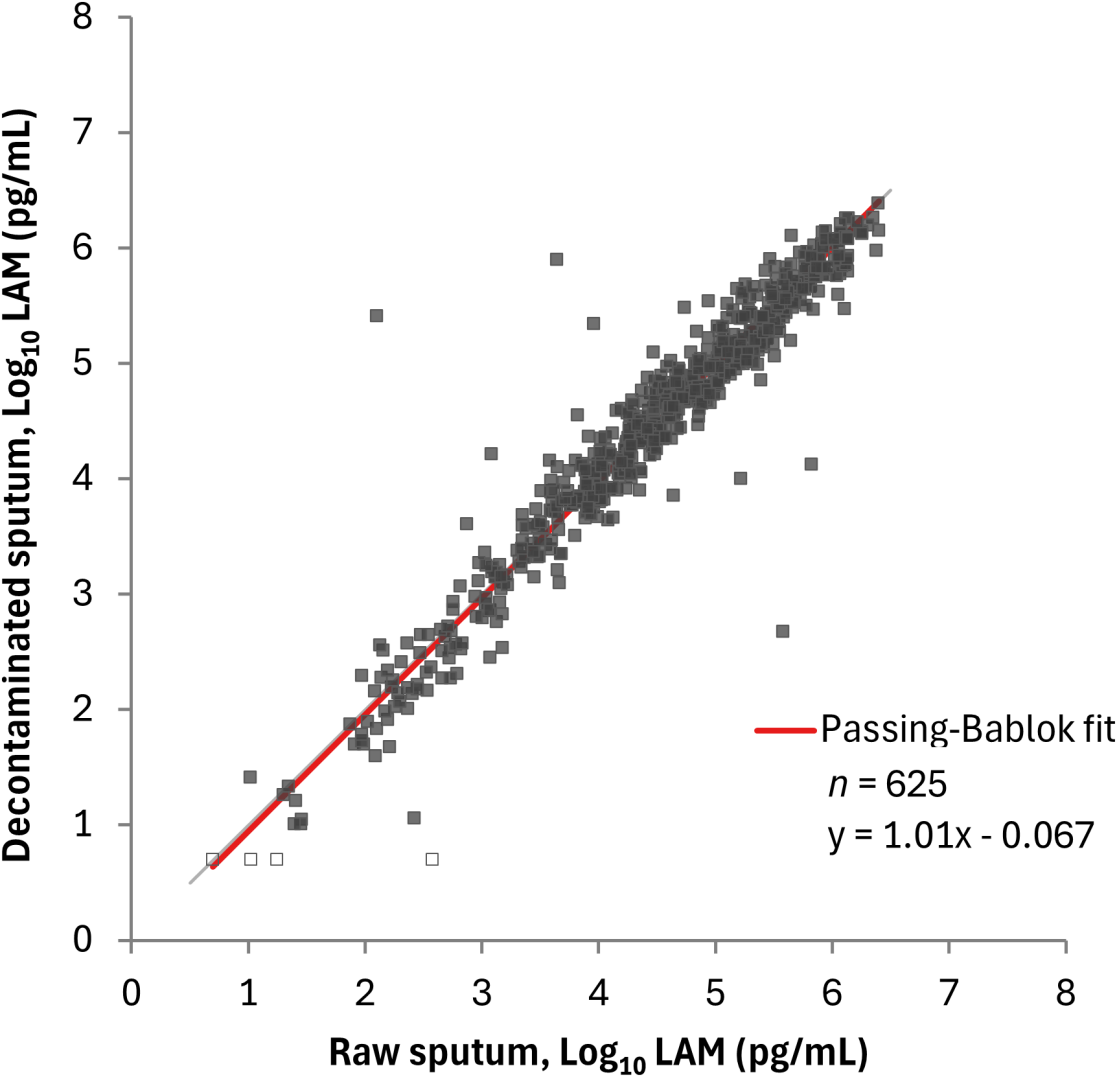
Correlation between LAM concentrations in raw sputum and decontaminated sputum by the PATHFAST-LAM. LAM concentrations below the lower limit of measurement were plotted as half of the lower limit of measurement (5.00 pg/mL for the PATHFAST-LAM) and represented open dots.

In the comparison between raw and decontaminated sputum using PATHFAST-LAM, Spearman’s correlation coefficient was 0.957 (95% CI: 0.950 – 0.964), with a slope of 1.01 (95% CI: 0.977 – 1.03) and an intercept of -0.067 (95% CI: -0.147 – 0.001) (**Figure 2**).

### Comparison between the PATHFAST-LAM and culture methods

**Figure 3:**
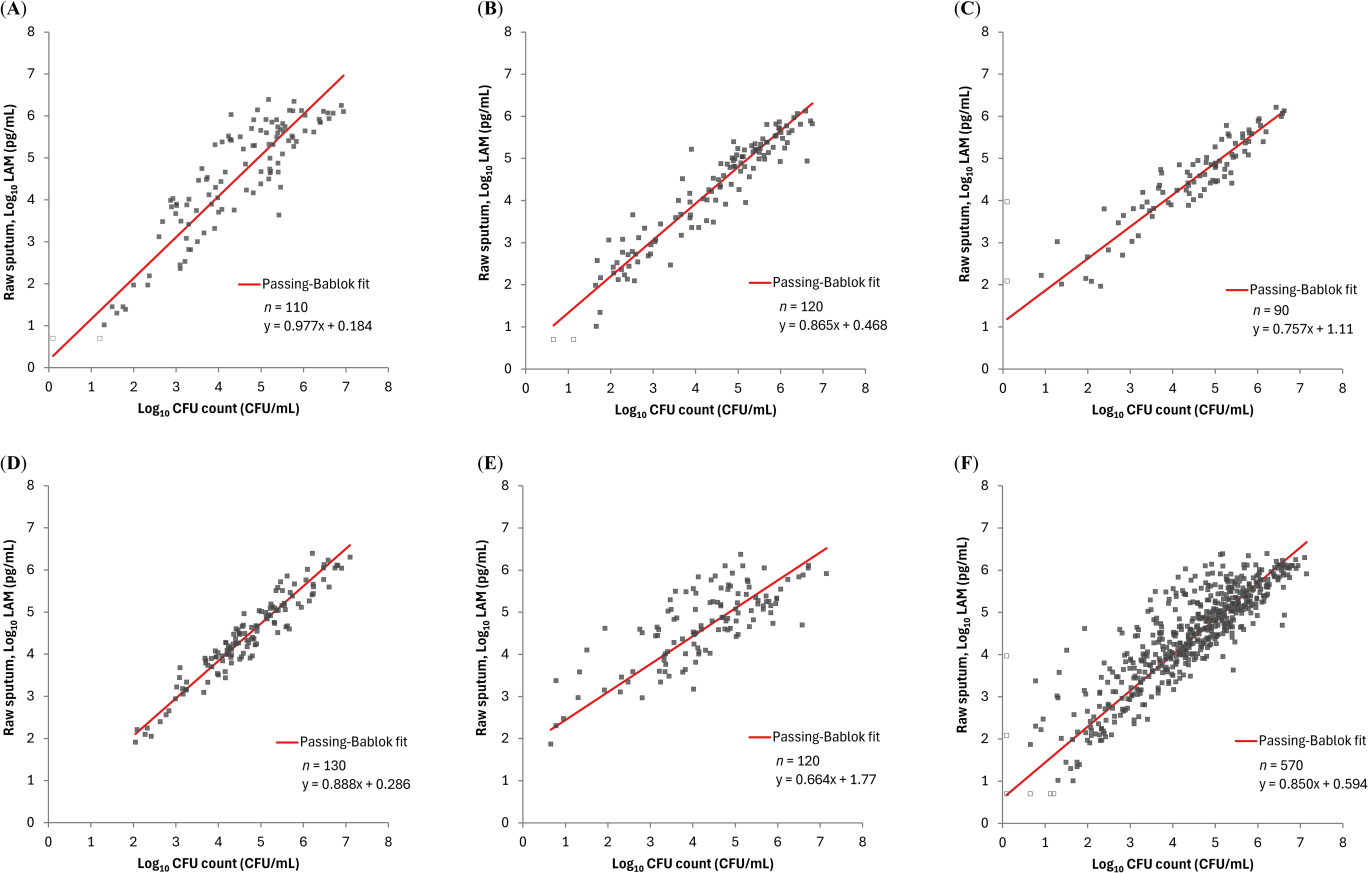
Relationship between LAM concentration by PATHFAST-LAM in raw sputum and CFU counts in groups of patients receiving different regimens LAM concentrations below the lower limit of measurement were plotted as half of the lower limit of measurement (Log_10_ 5.00 pg/mL) and represented as open dots. Similarly, negative CFU counts were plotted as Log_10_ 0.1 CFU/mL and also represented as open dots. **(A)** *r_s_* = 0.841 (95% CI: 0.773 – 0.889) for OPC-167832 3mg. **(B)** *r_s_* = 0.939 (95% CI: 0.912 – 0.957) for OPC-167832 10 mg. **(C)** *r_s_* = 0.921 (95% CI: 0.881 – 0.948) for OPC-167832 30 mg. **(D)** *r_s_* = 0.947 (95% CI: 0.925 – 0.963) for OPC-167832 90 mg. **(E)** *r_s_* = 0.723 (95% CI: 0.622 – 0.801) for RHEZ. **(F)** *r_s_* = 0.854 (95% CI: 0.830 – 0.876) for Overall.

We compared the PATHFAST-LAM results with CFU counts on solid culture and TTD from the MGIT system in each of the 5 treatment arms (OPC-167832 3 mg, 10 mg, 30 mg, 90 mg, and HRZE), and data from the 5 arms combined. The PATHFAST-LAM results in raw sputum showed a strong correlation with CFU counts across all five treatment arms, and combined data, with Spearman’s correlation coefficients ranging from 0.723 to 0.841 (**Figure 3**).

**Figure 4:**
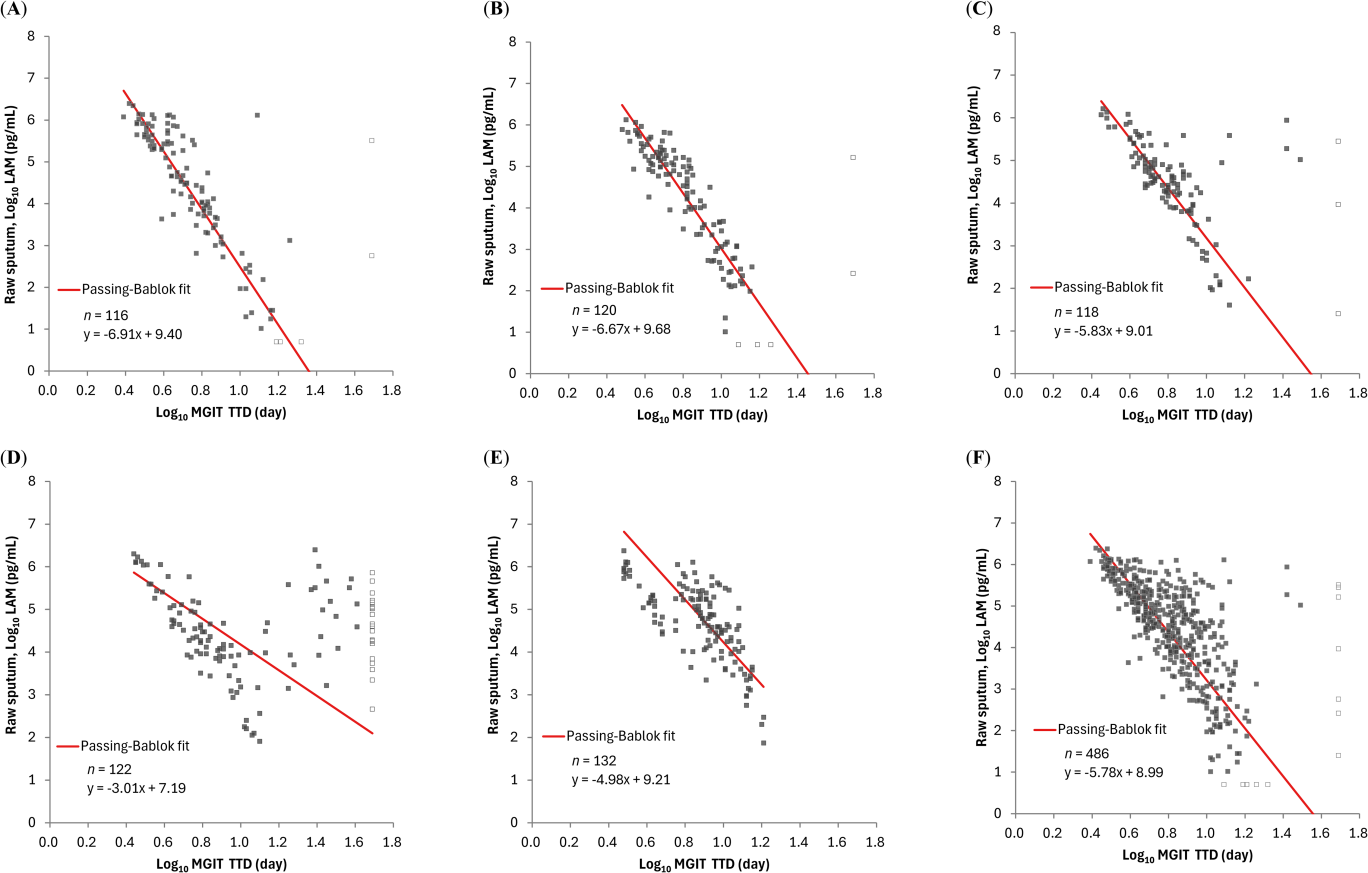
Relationship between LAM concentration by PATHFAST-LAM in raw sputum and TTD of MGIT in groups of patients receiving different regimens. LAM concentrations below the lower limit of measurement were plotted as half of the lower limit of measurement (Log_10_ 5.00 pg/mL) and represented open dots. Similarly, negative MGIT results were plotted as Log_10_ 49 day and are also represented as open dots. In all figures, MGIT results with positive but contaminated were excluded. **(A)** *r_s_* = -0.842 (95% CI: -0.889 – -0.778) for OPC-167832 3 mg. **(B)** *r_s_* = -0.885 (95% CI: -0.919 – -0.837) for OPC-167832 10 mg. **(C)** *r_s_* = -0.690 (95% CI: -0.777 – -0.579) for OPC-167832 30 mg. **(D)** *r_s_* = -0.251 (95% CI: -0.415 – -0.071) for OPC-167832 90 mg. **(E)** *r_s_* = -0.717 (95% CI: -0.793 – -0.620) for RHEZ. **(F)** *r_s_* = -0.727 (95% CI: -0.767 – -0.681) for Overall, excluding OPC-167832 90 mg.

Sputum LAM concentrations had negative correlations with TTDs: Spearman’s correlation coefficients ranged from -0.842 to -0.690 for the OPC-167832 3 mg, 10 mg, and 30 mg, respectively. The correlation was poor for the OPC-167832 90 mg cohort (Spearman’s correlation coefficient of -0.251) (**Figure 4**). As described in the original publication (Dawson et al., 2023), it was speculated that with 90 mg OPC-167832, drug carryover to the sample occurred at concentrations sufficient to prolong TTD. This reduces the correlation between CFUs or LAM concentrations with the TTD. Excluding the 90 mg cohort, LAM concentrations had a strong negative correlation with MGIT TTD as shown by the high correlation coefficient values.

To further evaluate the ability of PATHFAST-LAM to measure treatment response, we analyzed changes in PATHFAST-LAM results from raw sputum over the first 14 days of treatment, comparing them with CFU counts and MGIT TTD. On an individual patient level, the change in LAM concentrations during treatment were consistent with changes in CFU counts as showing in **Supplementary Material** (**Supplementary Figure S1**).

**Figure 5:**
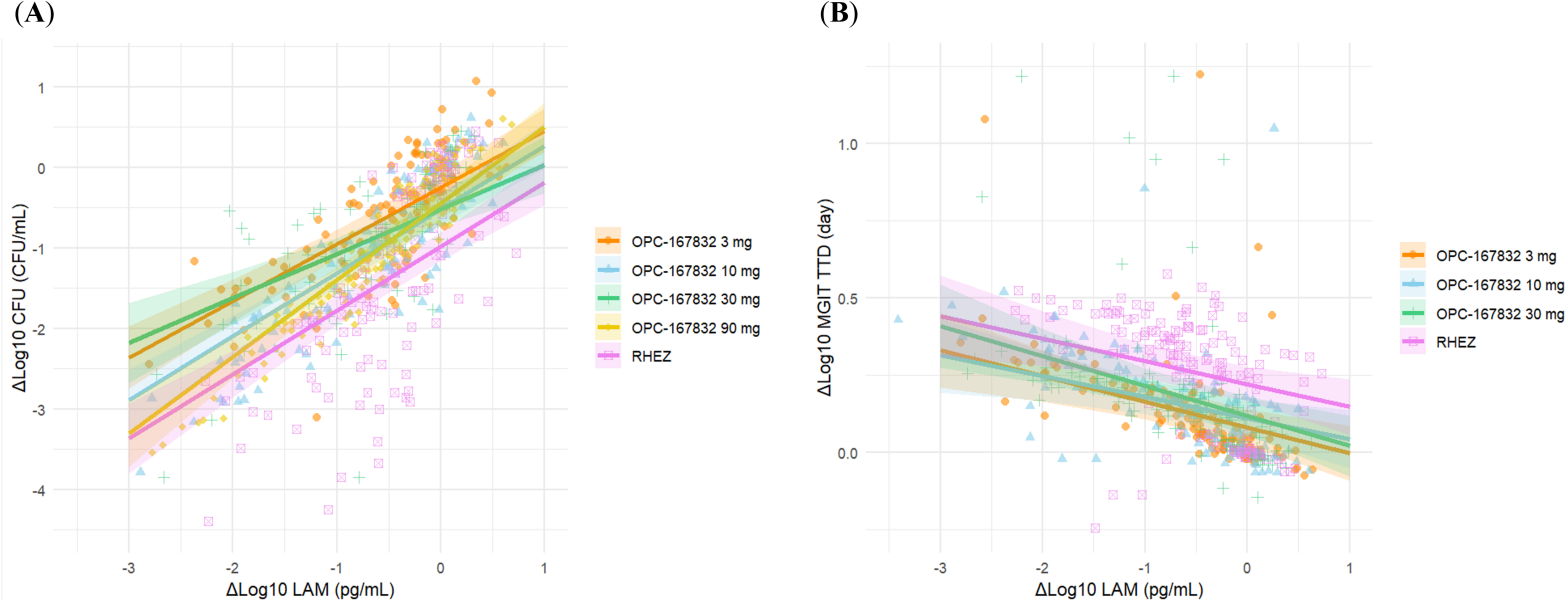
Estimated trends of ΔLog_10_ CFU and ΔLog_10_ MGIT in relation to ΔLog_10_ LAM by regimen. **(A)** Relationship between ΔLog_10_ CFU and ΔLog_10_ LAM by treatment regimen. **(B)** Relationship between ΔLog_10_ MGIT and ΔLog_10_ LAM by treatment regimen. Regression estimates were using a liner mixed-effects model with ΔLog_10_ LAM as a fixed effect and patient as a random effect. Shaded areas represented the 95% CI.

In addition, we evaluated the relationship between changes in LAM concentration (ΔLog_10_ LAM) and changes in CFU count (ΔLog_10_ CFU) using a linear mixed-effects model (**Figure 5**; **Table 1**). The interaction term (ΔLog_10_ LAM × regimen) allowed us to assess regimen-specific differences in the association between these biomarkers. The estimated slopes (ΔLog_10_ CFU per unit change in ΔLog_10_ LAM) varied by regimen, with values of 0.703 (OPC-167832 3 mg), 0.789 (OPC-167832 10 mg), 0.552 (OPC-167832 30 mg), 0.950 (OPC-167832 90 mg), and 0.793 (RHEZ). However, pairwise comparisons of slopes did not reveal significant differences between most regimens, except for the comparison between 30 mg and 90 mg (*p* = 0.0161). The reason for this detected difference between the 30 mg and the 90 mg is unknow, but is unlikely related to the drug mechanism as there is no difference between the 90 mg and the 3 mg, or the 10 mg. For MGIT TTD, a similar analysis was performed using the same model framework, excluding the OPC-167832 90 mg regimen. The estimated slopes for MGIT were more consistent across regimens, with values ranging from -0.0674 (OPC-167832 10 mg) to -0.0970 (OPC-167832 30 mg). No significant pairwise differences were observed (all *p* > 0.9).

**Table 1:** Estimated slopes (95% CI) for the association between ΔLog_10_ LAM and ΔLog_10_ CFU or ΔLog_10_ MGIT by regimen.

Regimen	ΔLAM vs. ΔCFU Slope (95% CI)	ΔLAM vs. ΔMGIT Slope (95% CI)
OPC-167832 3 mg	0.703 (0.560 – 0.845)	-0.0835 (-0.128 – -0.0386)
OPC-167832 10 mg	0.789 (0.645 – 0.932)	-0.0674 (-0.114 – -0.0206)
OPC-167832 30 mg	0.552 (0.361 – 0.743)	-0.0970 (-0.149 – -0.0453)
OPC-167832 90 mg	0.950 (0.788 – 1.11)	N.A.
RHEZ	0.793 (0.633 – 0.952)	-0.0734 (-0.123 – -0.0236)

Estimates were obtained using a linear mixed-effects model with ΔLAM as a fixed effect and patient as a random effect.

### Missing data rate

Contamination, a common issue in culture-based methods, can prevent quantitative analysis of viable bacterial load. We examined the missing data rate of the PATHFAST-LAM compared with culture-based methods. Among the samples tested, the PATHFAST-LAM demonstrated a system failure rate of 0.0% for both raw sputum (0/634) and decontaminated sputum (0/637).

In contrast, the solid culture for CFU counts showed 9.6% (62/649) missing data, including 52 cases lost due to contamination on both plates, 9 cases classified as unknown on both plates, and 1 with other reasons. Separately, 0.5% (3/649) showed no MTB variants growth on both plates but tested positive in MGIT.

The MGIT system had 4.2% (27/649) of cases with incomplete TTD data, including 2 cases lost due to contamination and 25 cases positive for MTB variants but contaminated. Additionally, 4.5% (29/649) showed no MTB variants growth; of these, 27 were positive in CFU counts, and 2 had unknown CFU results.

### Turnaround time

In this MAD/EBA trial, CFU counts were obtained from agar media and required culture for up to 3 weeks. In this trial, the average TTD is 4-5 days prior to treatment and progressively became longer during treatment up to the 42-day cutoff. While LAM measurement by the PATHFAST-LAM was conducted on batched samples in this study, a measurement on a sample can be obtained within one hour after receiving the specimen. Therefore, the PATHFAST-LAM can be a significant time saving.

## Discussion

This study represents an initial validation of the PATHFAST-LAM within an EBA trial, focusing on its performance for TB treatment monitoring over the first 14 days of therapy. Our results suggest that LAM measured by the PATHFAST-LAM has potential as a bacterial load-reflecting biomarker. This finding highlights its utility as an alternative to conventional culture-based methods for TB treatment monitoring. Notably, the PATHFAST-LAM offers faster, safer, and more accessible testing, making it valuable in both routine TB care and drug development.

Previous studies on the LAM-ELISA have demonstrated that LAM concentration in sputum correlates well with CFU counts and MGIT TTD, using samples from TB patients collected from pre-treatment up to a maximum of 56 days post-treatment initiation (Kawasaki et al., 2019; Jones et al., 2022; Dawson et al., 2023). In our study, the PATHFAST-LAM showed a strong correlation with the LAM-ELISA in both raw and decontaminated sputum samples (**Figure 1**), suggesting comparable performance for treatment monitoring. Furthermore, LAM concentrations measured by the PATHFAST-LAM strongly correlated with both CFU counts and MGIT TTD across treatment arms (**Figures 3**, **4**). The individual patient data (**Supplementary Figure S1**) also showed consistent longitudinal trends between LAM, CFU, and MGIT TTD, supporting their potential usefulness for monitoring bacterial load dynamics over time.

Although mixed-effects model analysis revealed regimen-dependent differences in the slopes of relationship between ΔLAM and ΔCFU or ΔMGIT (**Figure 5** and **Table 1**), the overall similarity in ΔLAM trends across regimens suggests that LAM could serve as a practical alternative to CFU or MGIT without requiring regimen-specific adjustments. This consistency enhances its potential utility in treatment monitoring, as LAM measurements would not need to be corrected for different drug regimens. However, some regimen-dependent variability was observed, and it might be attributable to differences in the mechanisms of action of the drugs used, particularly those affecting mycobacterial cell wall integrity, such as isoniazid (Winder and Collins, 1970), ethambutol (Takayama and Kilburn, 1989; Deng et al., 1995), and OPC-167832 (Hariguchi et al., 2020). Another possible contributing factor is the impact of treatment on bacterial subpopulations, including viable but non-culturable (VBNC) bacteria (Bowness et al., 2015; Barr et al., 2016; Mishra and Saito, 2022). While CFU count primarily reflects culturable bacteria, LAM levels may also capture contributions from metabolically active but non-culturable populations. This could explain why CFU decline did not always align proportionally with LAM reduction across regimens.

Additionally, Dawson et al. reported drug carryover effects, particularly in the 90 mg OPC-167832 cohort, with an impact on MGIT TTD (Dawson et al., 2023). Failing to account for drug carryover in culture may lead to an overestimation of drug efficacy. This may be concerning for drugs with very low MICs such as OPC-167832. However, the drug carryover effects are not expected to impact the LAM measurement. Previous research, such as an analytical performance study by Akinaga et al., indicates that the PATHFAST-LAM showed no interference from major anti-TB drugs up to 100 µg/mL (Akinaga et al., 2024). This suggests a potential robustness of the assay’s consistency and reliability in monitoring treatment response, even in instances where drug carryover could be an issue impacting the culture results.

Our study also highlighted the advantage of PATHFAST-LAM vs. culture-based methods in data reliability and robustness. Culture-based methods demonstrated higher rates of data loss due to contamination and other issues, a problem that is reportedly more prevalent in routine practice in LMICs (Okumu et al., 2017; Mohammed Adam et al., 2022). This data loss is also recognized as a challenge in drug development (Gillespie et al., 2024). In our study, missing data rates were 9.6% (62/649) for CFU counts and 4.2% (27/649) for MGIT. While the contamination rate in solid culture slightly exceeded the commonly reported ranges of 3 – 5% (European center for disease prevention and control (ECDC), 2023), data loss was observed at multiple points in some patients, suggesting that patient-specific factors rather than procedural errors may have contributed to the issue, and the rate was therefore considered acceptable. In contrast, the PATHFAST-LAM provided a 0.0% missing data rate in either raw (0/634) or decontaminated sputum samples (0/637), demonstrating remarkable reliability. Although this assessment did not consider potential failure in the sample pre-treatment process, this reliability makes PATHFAST-LAM particularly advantageous for longitudinal patient monitoring.

Despite these promising findings, several limitations remain. First, while TB treatment is a long-term process, this study analyzed data only from the first 14 days of treatment, highlighting the need for further investigation into the utility of PATHFAST-LAM in long-term monitoring, as outlined in the TPPs or guideline for TB treatment monitoring (World Health Organization (WHO), 2023; Gillespie et al., 2024; MacLean et al., 2024). A key consideration is that, later in treatment, when bacterial load is lower, culture may remain positive while LAM levels decline due to sensitivity limitations. Our study did not evaluate this scenario, which warrants further investigation.

Second, although the PATHFAST-LAM showed a good correlation with CFU counts and MGIT TTD, mixed-effects model analysis revealed some differences in the relationship between ΔLog_10_ LAM and ΔLog_10_ CFU or ΔLog_10_ MGIT across treatment regimens. Further studies are required to clarify whether these differences reflect variations in bacterial killing, LAM release kinetics, or other biological factors influencing LAM detectability.

Third, this study used frozen LAM extract samples rather than fresh sputum samples. While potential differences between fresh and frozen samples cannot be entirely ruled out, internal study data from the PATHFAST-LAM demonstrated that LAM levels remain stable for at least nine months under frozen conditions. Furthermore, in this study, PATHFAST-LAM results from frozen samples showed a good correlation with LAM-ELISA results, which were obtained from comparatively fresher samples (**Figure 1**). Therefore, sample freezing on LAM measurements may be limited. However, this observation may not generalize to all settings, and further studies using prospectively collected, fresh samples are warranted to confirm these results.

Lastly, while the PATHFAST-LAM demonstrated a 0.0% missing data rate in our study, several challenges remain for its implementation in LMIC settings. The validity of the assay in these settings may be significantly influenced by factors such as sample collection, pre-treatment steps, staff training, and resource allocation, which were not fully evaluated here. Although the assay offers practical advantages over LAM-ELISA and culture methods, its overall cost-effectiveness remains unclear. Beyond the test price, factors such as analyzer maintenance, workflow efficiency, staff workload, and healthcare resource optimization should be considered to assess whether the PATHFAST-LAM can be a sustainable option in LMICs. Future studies are essential to comprehensively evaluate these factors.

Regarding biosafety, LAM extraction in this study was conducted under biosafety level 3 (BSL-3) conditions, as required for handling sputum potentially containing viable MTB, and PATHFAST measurements were conducted under biosafety level 2 (BSL-2) conditions, in accordance with applicable safety standards. However, in many LMICs, tests such as smear microscopy or GeneXpert are already performed under lower biosafety levels, often with simplified precautions. The PATHFAST-LAM assay involves a heat and alkaline treatment step during sample pre-processing, which may reduce the risk of viable pathogen exposure to a level comparable to that of smear microscopy or GeneXpert. The risk mitigation provided by sample pre-treatment may allow the assay to be safely deployed under conditions lower than BSL3, using the same precautions as those already applied for these frontline diagnostic tools in real-world settings.

In conclusion, our findings indicate that the PATHFAST-LAM has the potential as a monitoring tool for TB treatment. Its ability to provide rapid, reliable data without culture-related failure makes it particularly promising for early treatment assessment. However, further studies are needed to validate its long-term performance, clarify regimen-dependent variations, and evaluate its performance in prospective trials using fresh samples, particularly in LMIC settings, to assess its robustness and real-world applicability.

## Data Availability

The original contributions presented in the study are included in the article/**Supplementary Material**. Further inquiries can be directed to the corresponding author/s.
