# Supplementary material for: Quantification of bactericidal activity using the PATHFAST TB LAM Ag assay during the first 14 days of pulmonary tuberculosis treatment
Source: Front Antibiot. 2025 May 15;4:1574688. doi: 10.3389/frabi.2025.1574688 (PMC12120838; doi:10.3389/frabi.2025.1574688)
Supplement: Supplementary file 1 [file DataSheet1.docx]

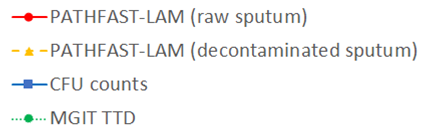


(**A**) OPC-167832 3 mg


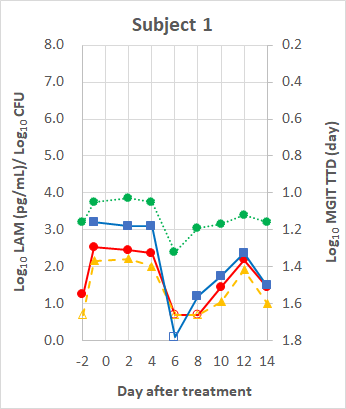

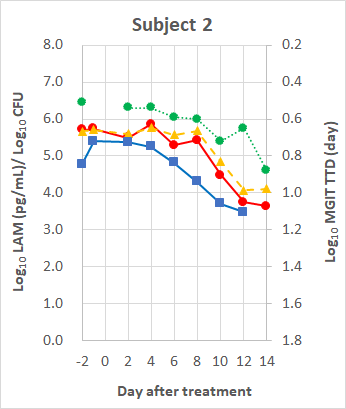

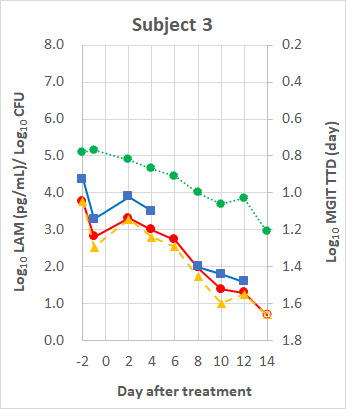

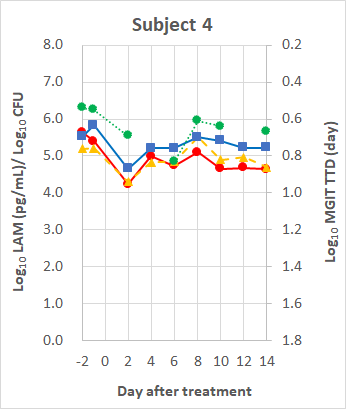

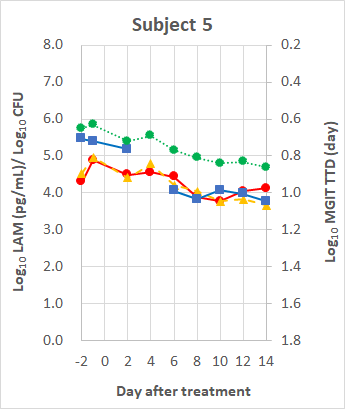


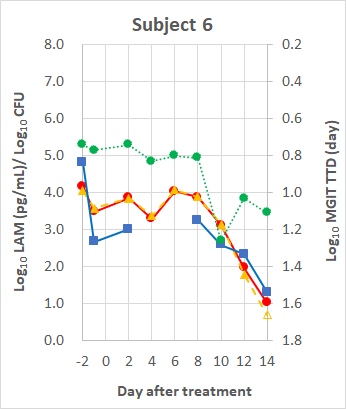

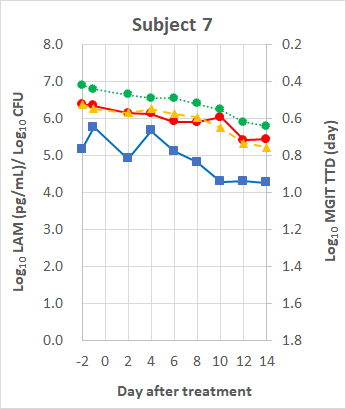

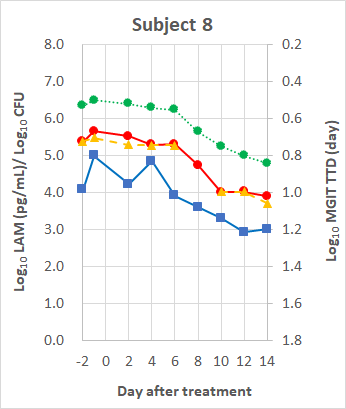

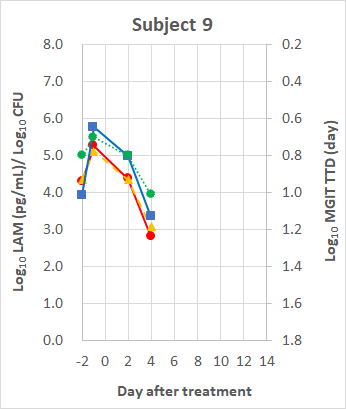

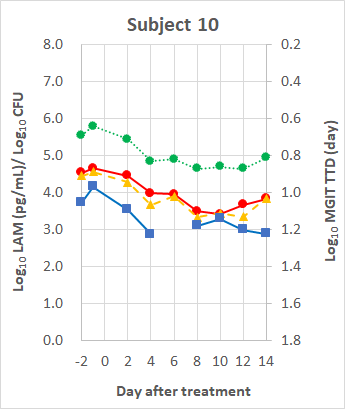


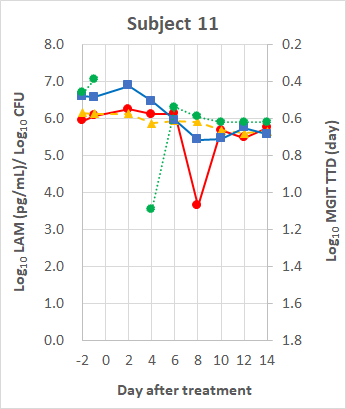

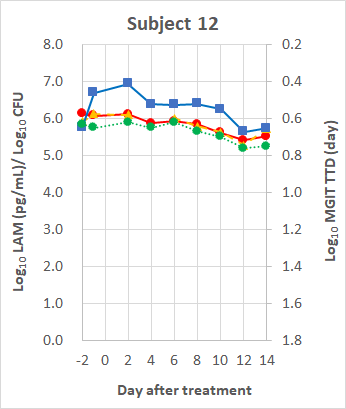

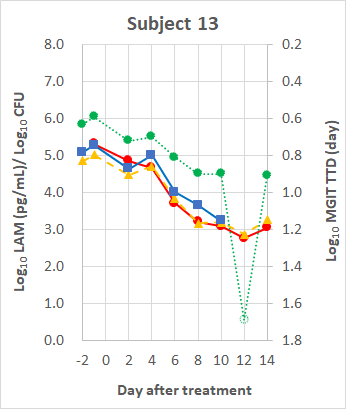

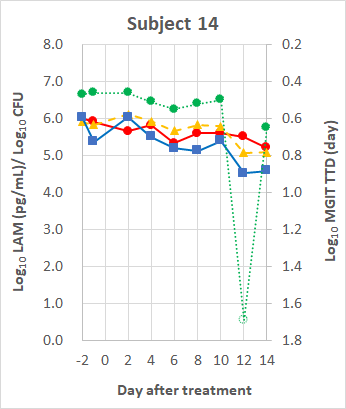


**Fig. S1. Test results for individual patient during treatment (data from 75 patients).** LAM concentrations below the lower limit of measurement were plotted as half of the lower limit of measurement (Log_10_ 5.00 pg/mL) and represented with open dots. Zero CFU counts were plotted as Log_10_ 0.1 CFU/mL and represented with open dots. MGIT-negative results were plotted as Log_10_ 49 days and similarly represented with open dots. In all figures, MGIT results with positive but contaminated were excluded. (**A**) OPC-167832 3 mg. (**B**) OPC-167832 10 mg. (**C**) OPC-167832 30 mg. (**D**) OPC-167832 90 mg. (**E**) RHEZ.


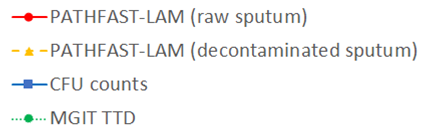


(**B**) OPC-167832 10 mg


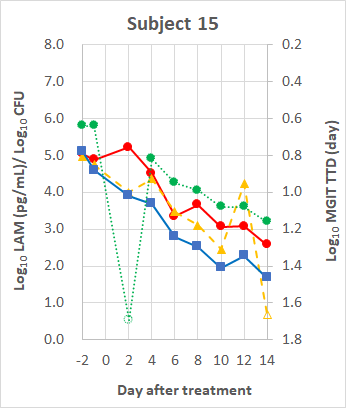

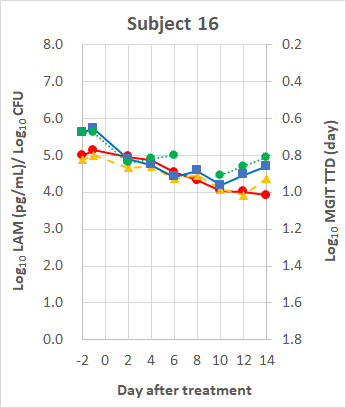

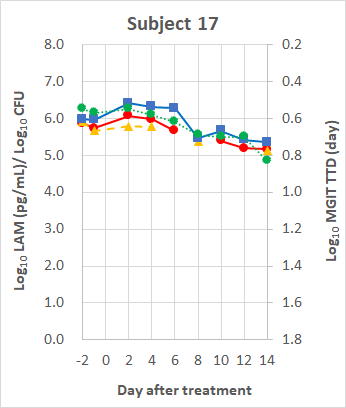

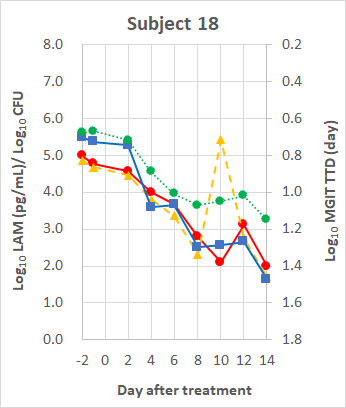

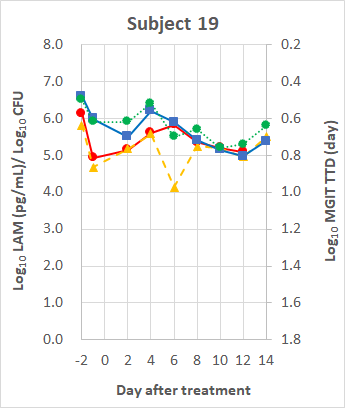


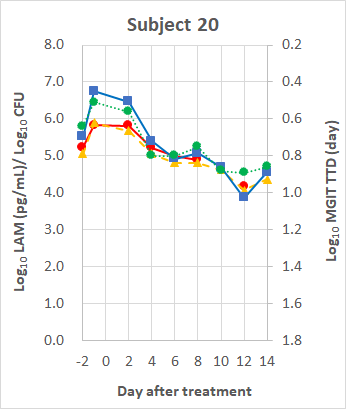

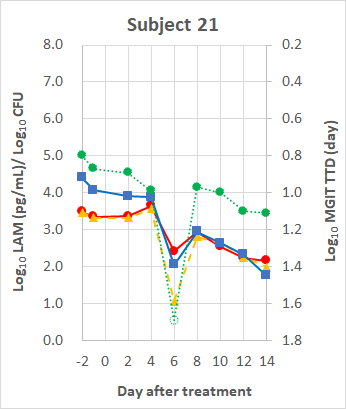

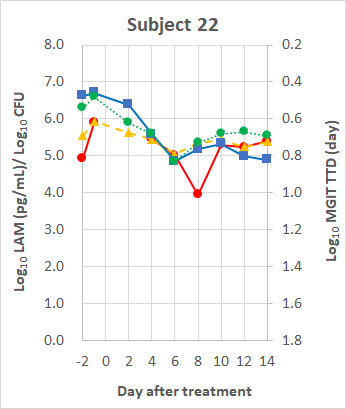

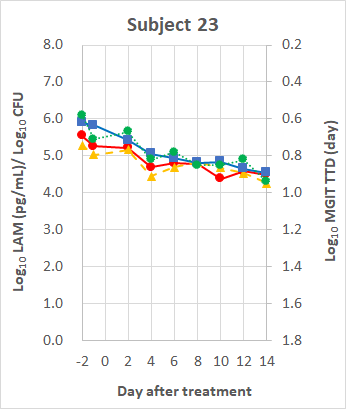

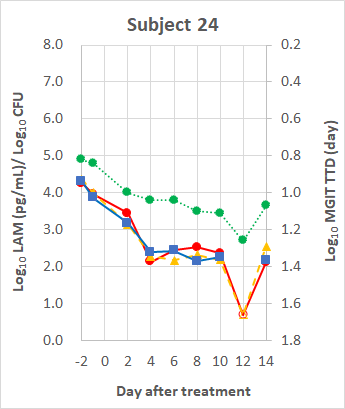


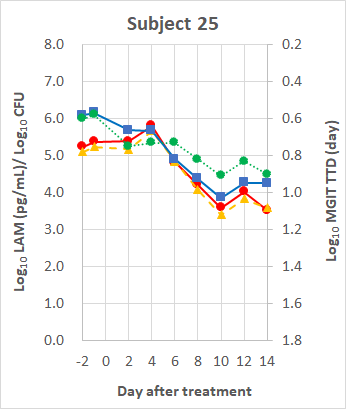

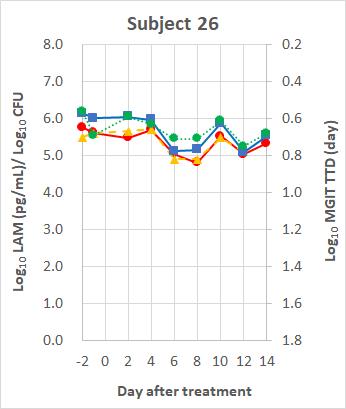

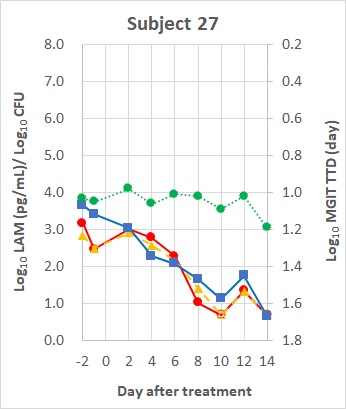

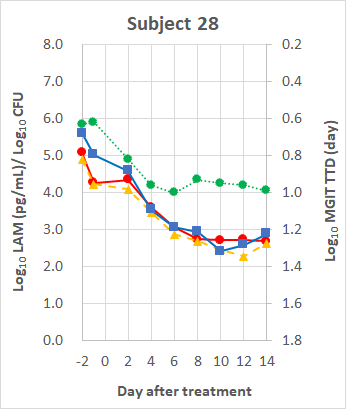


**Fig. S1. Continued. Test results for individual patient during treatment (data from 75 patients).**


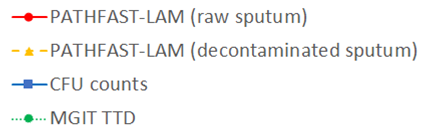


(**C**) OPC-167832 30 mg


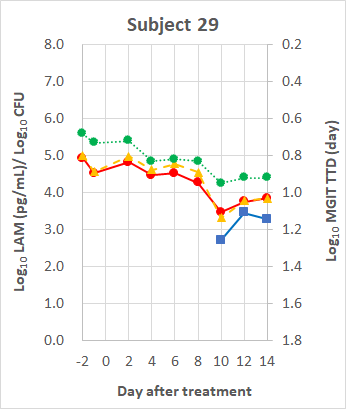

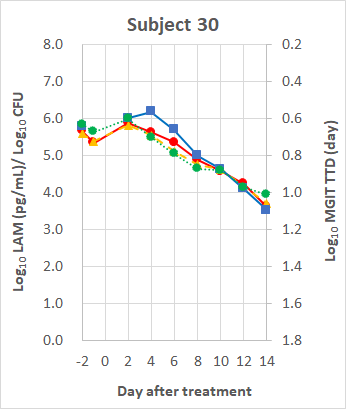

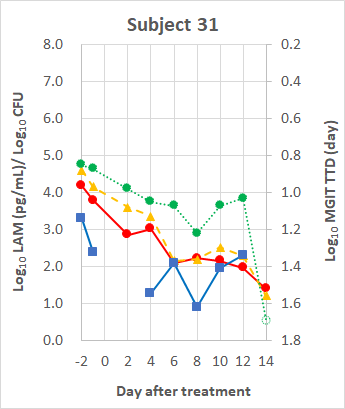

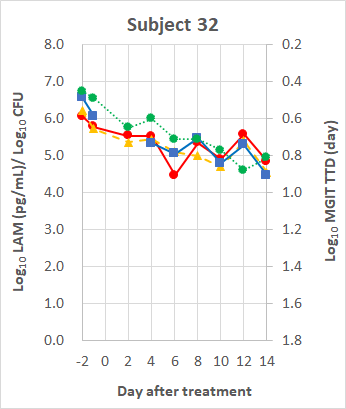

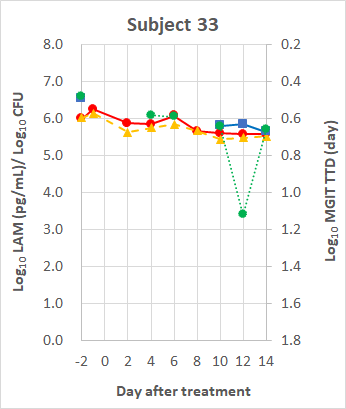


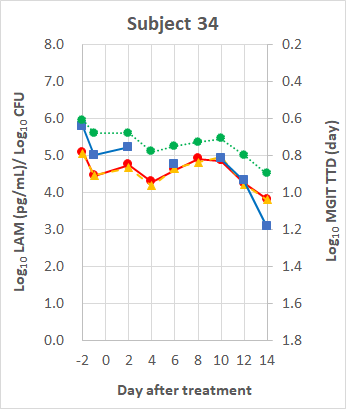

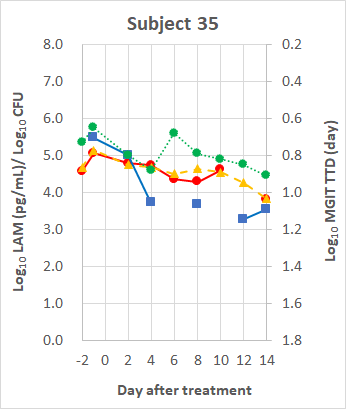

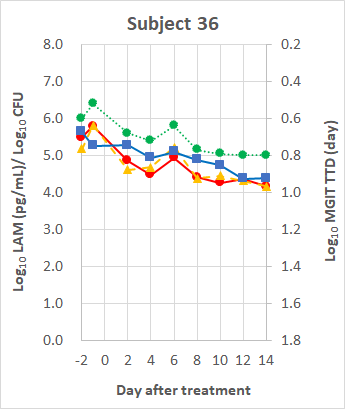

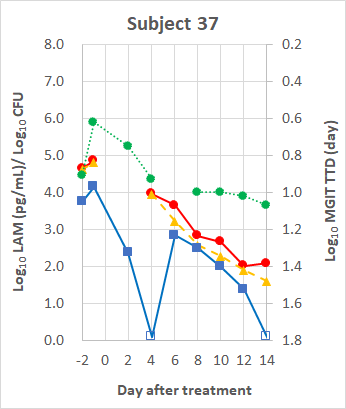

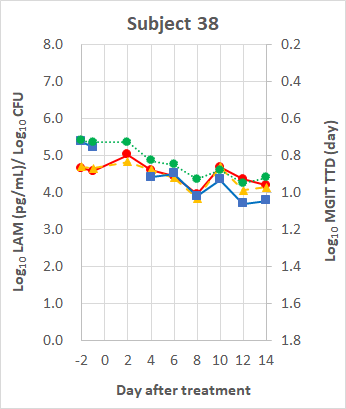


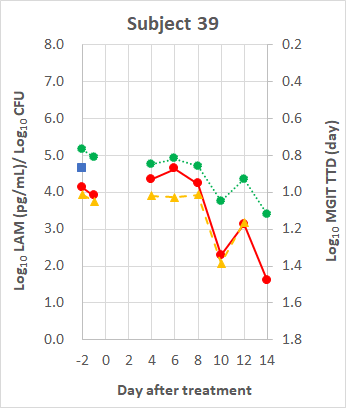

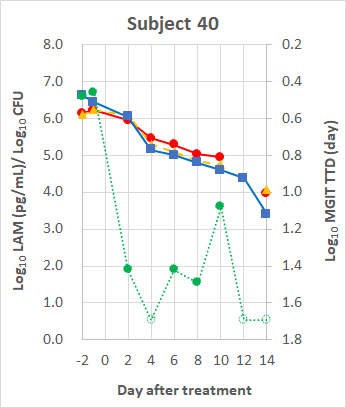

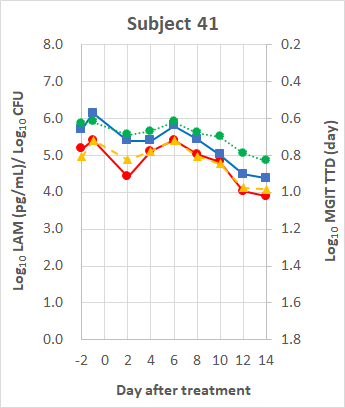

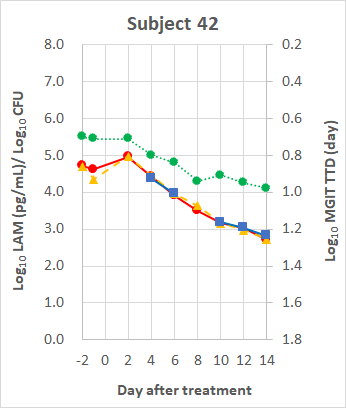


**Fig. S1. Continued. Test results for individual patient during treatment (data from 75 patients).**


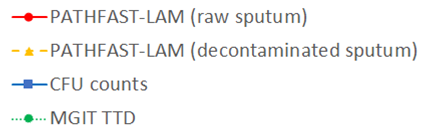


(**D**) OPC-167832 90 mg


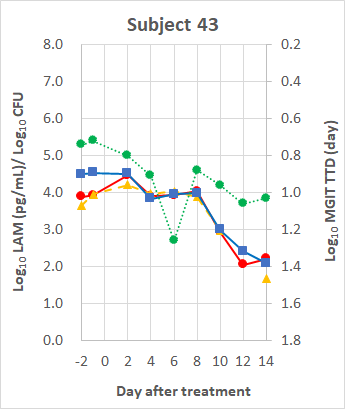

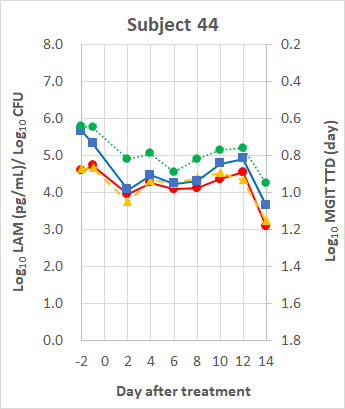

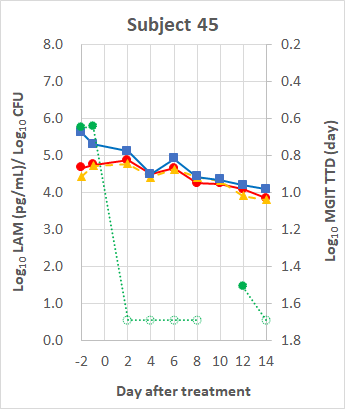

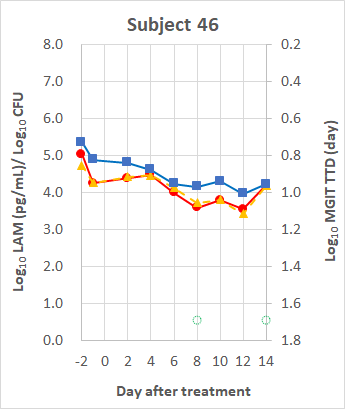

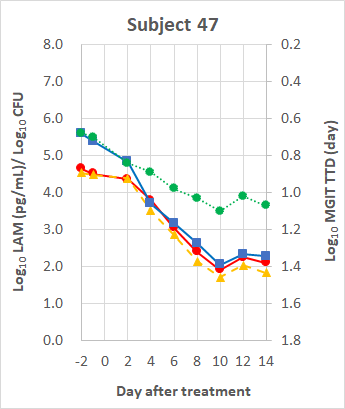


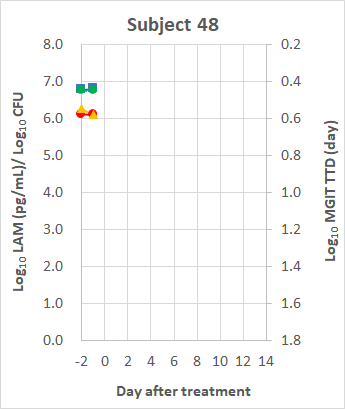

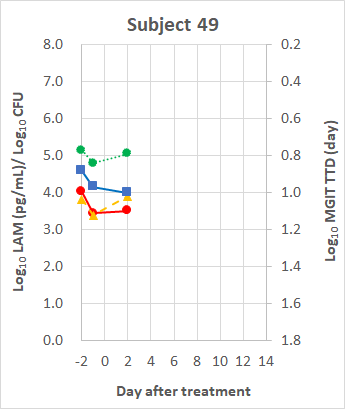

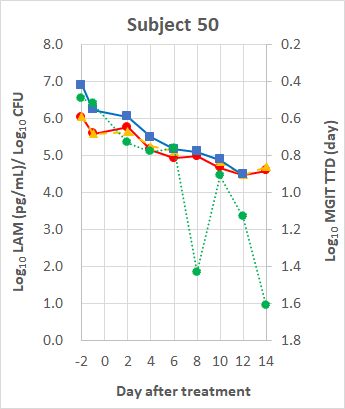

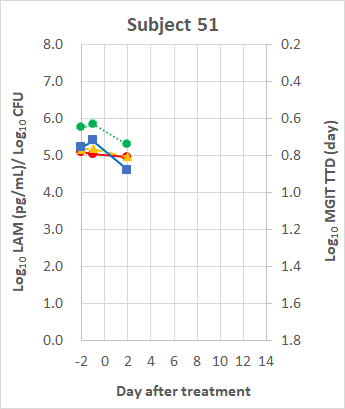

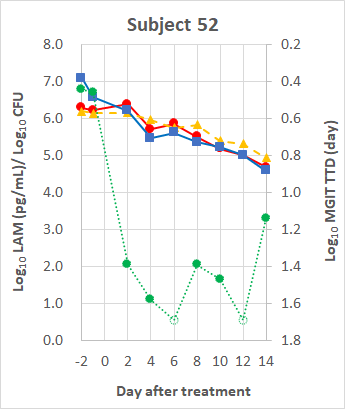


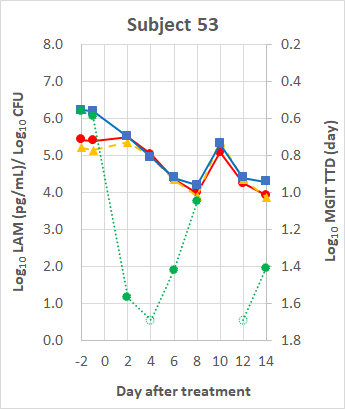

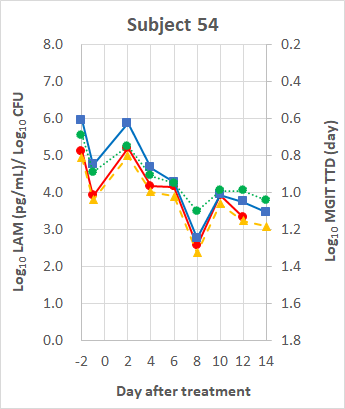

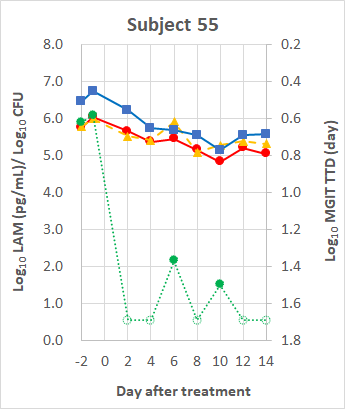

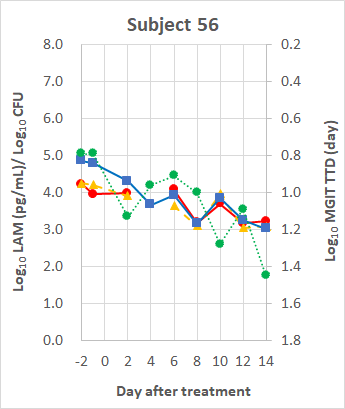

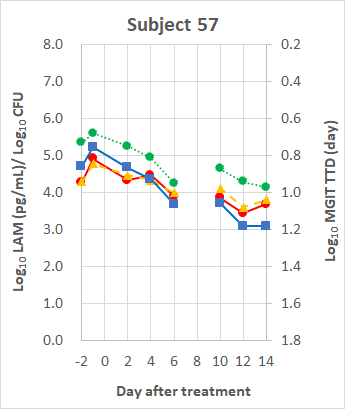


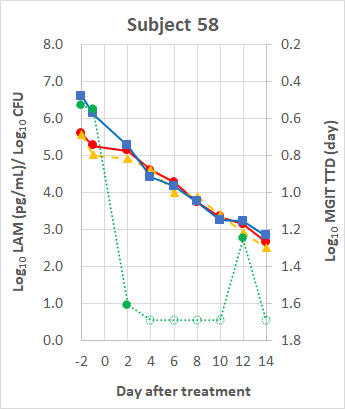

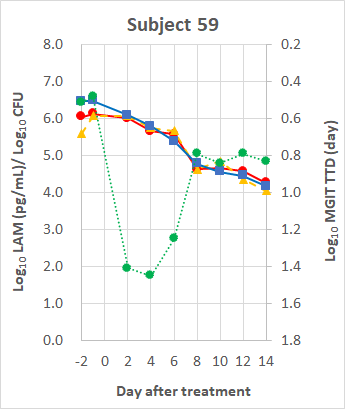


**Fig. S1. Continued. Test results for individual patient during treatment (data from 75 patients).**


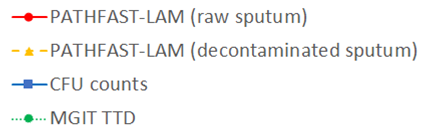


(**E**) RHEZ


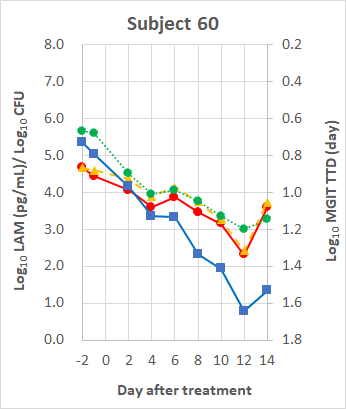

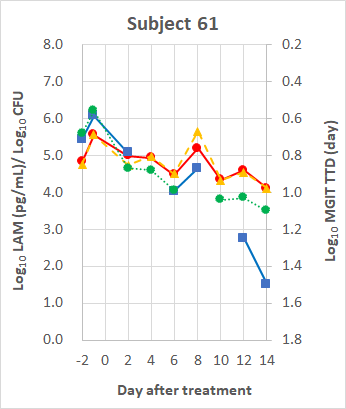

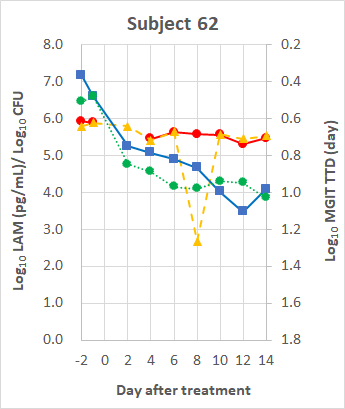

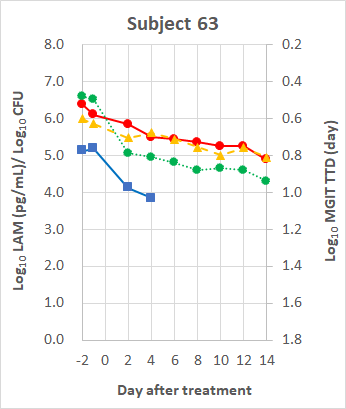

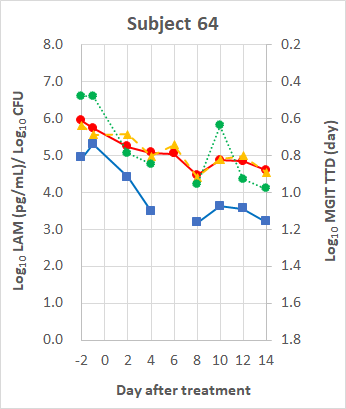


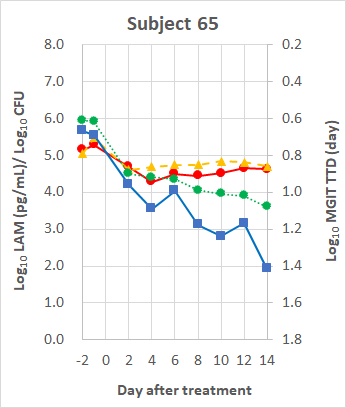

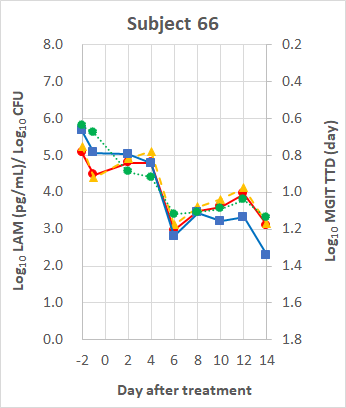

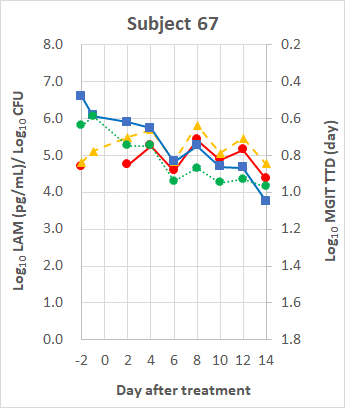

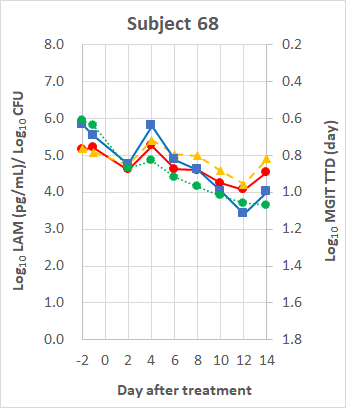

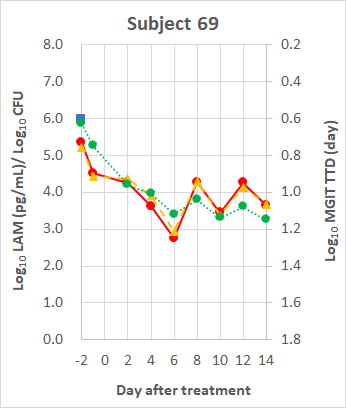


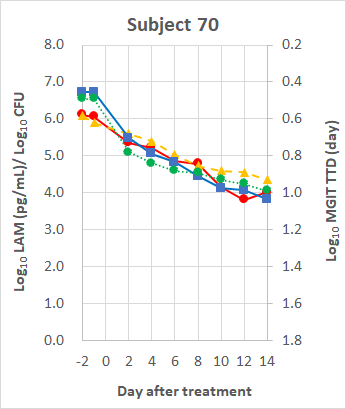

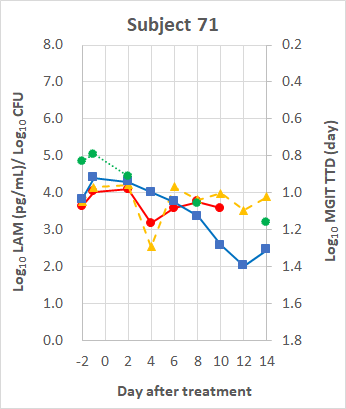

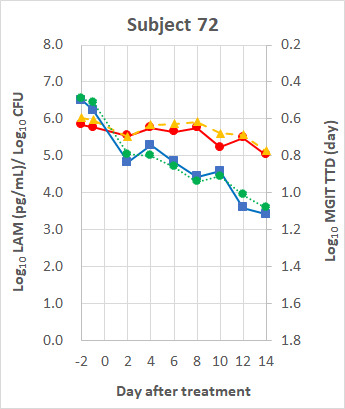

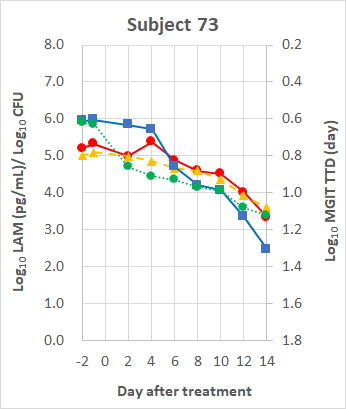

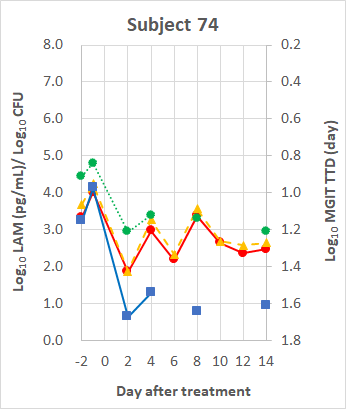


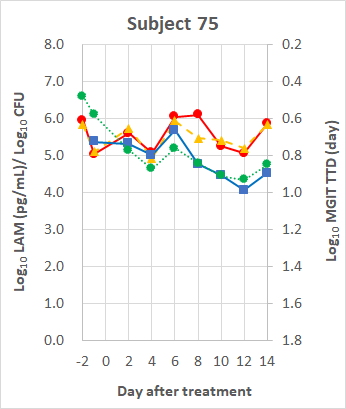


**Fig. S1. Continued. Test results for individual patient during treatment (data from 75 patients).**
